# Oxygen vacancies induced photoluminescence in $\hbox {SrZnO}_2$ nanophosphors probed by theoretical and experimental analysis

**DOI:** 10.1038/s41598-020-74436-8

**Published:** 2020-10-15

**Authors:** Manju, Megha Jain, Saibabu Madas, Pargam Vashishtha, Parasmani Rajput, Govind Gupta, Mousumi Upadhyay Kahaly, Kemal Özdoğan, Ankush Vij, Anup Thakur

**Affiliations:** 1 Advanced Materials Research Lab, Department of Basic and Applied Sciences, Punjabi University Patiala, Patiala, Punjab 147 002 India; 2 Department of Physics, Punjabi University Patiala, Patiala, Punjab 147 002 India; 3 ELI-ALPS, ELI-HU Non-Profit Ltd., Wolfgang Sandner utca 3., H-6728 Szeged, Hungary; 4 Institute of Physics, University of Szeged, Dóm tér 9, 6720 Szeged, Hungary; 5 Sensor Devices and Metrology Group, CSIR-National Physical Laboratory (CSIR-NPL), Dr. K. S. Krishnan Road, New Delhi, 110 012 India; 6 Academy of Scientific and Innovative Research, (AcSIR), CSIR-HRDC Campus, Ghaziabad, Uttar Pradesh 201 002 India; 7 Atomic and Molecular Physics Division, Bhabha Atomic Research Center, Trombay, Mumbai, 400 085 India; 8 Department of Physics, Yildiz Technical University, 34210 Istanbul, Turkey; 9 Nanophosphors Lab, Department of Physics, Amity University Haryana, Gurgaon, Haryana 122 413 India; 10 Department of Physics, University of Petroleum and Energy Studies, Dehradun, Uttarakhand 248 007 India

**Keywords:** Materials for optics, Nanoscale materials, Theory and computation, Electronic structure

## Abstract

We report, for the first time, the influence of oxygen vacancies on band structure and local electronic structure of $\hbox {SrZnO}_2$ (SZO) nanophosphors by combined first principle calculations based on density functional theory and full multiple scattering theory, correlated with experimental results obtained from X-ray absorption and photoluminescence spectroscopies. The band structure analysis from density functional theory revealed the formation of new energy states in the forbidden gap due to introduction of oxygen vacancies in the system, thereby causing disruption in intrinsic symmetry and altering bond lengths in SZO system. These defect states are anticipated as origin of observed photoluminescence in SZO nanophosphors. The experimental X-ray absorption near edge structure (XANES) at Zn and Sr *K*-edges were successfully imitated by simulated XANES obtained after removing oxygen atoms around Zn and Sr cores, which affirmed the presence and signature of oxygen vacancies on near edge structure.

## Introduction

Oxide systems are widely explored as functional materials^1–3^ and have been a playground for both fundamental as well as application based research because of exhibiting interesting properties attributed by native point defects. Presence of oxygen vacancies imparts interesting effects on optical, magnetic and structural properties in oxide semiconducting materials such as ZnO, $\hbox {PbTiO}_3, \hbox {SrTiO}_3$, $\hbox {ZnAl}_2\hbox {O}_4, \hbox {SnO}_2$^2,4–10^. The wide band gap $\hbox {SrZnO}_2$ (SZO) nanophosphors, when excited with suitable energy of photons, are reported to be emitting interesting luminescence upon doping with various luminescent activators^11–13^. The undoped state of SZO nanophosphors exhibits broad emission in visible region^14,15^, which could be resulting from below band gap states created by lattice defects. Thermoluminescence analysis on SZO nanophosphors upon gamma irradiation revealed that various shallow and deep defect levels are present in the forbidden gap^16^. The absence of suitable theoretical and experimental characterizations identifying defect structure of SZO has created lacunae in the study on this material.

The presence of lattice defects^17^, for instance oxygen vacancies, has deep impact on band structure as well as on local electronic structure of the material. Numerous experimental techniques such as photoluminescence (PL), electron spin resonance, magnetic characterizations^4,18,19^ etc. have been used to characterize oxygen vacancies in various systems. A significant amount of interest is also shown by theoretical researchers in studying structural model with oxygen vacancies, for which density functional theory (DFT) based calculations are widely trusted and employed^20–23^. The oxygen vacancy alters the properties of oxide materials, by creating F centres in ionic oxides, causing the formation of metal-metal bonds in covalent oxides or altering the oxidation state of compounds having transition metal atoms with empty *d* orbitals and hence ultimately affecting the local electronic structure of the linked atoms^24^. X-ray absorption spectroscopy (XAS) is highly efficient technique which probes the density of unoccupied states of element of interest and provides the information about oxidation state, interatomic bond distances and coordination number of the absorber atom^25,26^.

In context of SZO system, there is no study of structural model with oxygen vacancies as well as local electronic study in this system justifying the luminescence exhibited by SZO nanophosphors. In this work, we have presented a panoramic view by covering role of oxygen vacancies on band structure and local electronic structure to its application in lighting devices. The band structure for pure as well as oxygen vacancy incorporated system is simulated by DFT calculations. To probe the effect of vacancy on local electronic structure, XAS study at Zn and Sr absorbing sites is performed. The qualitative analysis of X-ray absorption near edge structure (XANES) is done by real space full multiple scattering simulations to get best match of theory and experimental results. The consequence of oxygen vacancies in the system is seen as broad band PL spectrum, centred at $\sim$510 nm, exhibited by SZO nanophosphors.

## Results and discussion

**Figure 1:**
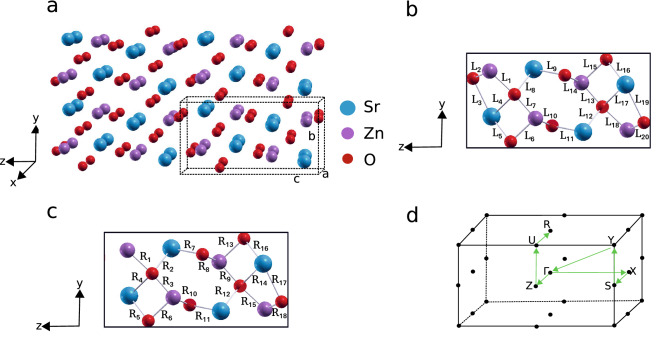
Lattice structure of SZO: (**a**) Crystal structure of SZO marked with the coordinate axes (x, y, z) and lattice vectors a = 3.3748 Å, b = 5.9179 Å, and c = 11.4575 Å. Sr, Zn, and O atoms are represented with blue, purple, and red colored spheres, respectively. (**b**) Unit cell (projected along x-direction) of SZO and associated bond lengths ($\hbox {L}_{1-20}$). (**c**) Unit cell of SZO with 12.5% O vacancy, and modified bond lengths, represented by R${_{1-18}}$. (**d**) Irreducible Brillouin zone with high symmetry points.

SZO belongs to orthorhombic family with each unit cell having four formula units. The crystal structure with lattice vectors *a*, *b*, *c* is shown in Fig. 1(a). The unit cell of SZO, which is represented by dashed lines, consists of 16 atoms (four Sr, four Zn and eight O) in total (Fig. 1(b)). Our optimised lattice constants $a =3.3748$ Å, $b = 5.9179$ Å, and $c = 11.4575$ Å are well in agreement with our experimental results^16^ and that reported in literature^27^.

The atomic arrangement in SZO lattice is not like that for regular systems reported so far in literature. Zn and Sr are coordinated with 4 and 7 oxygen atoms, respectively. There are two types of oxygen in system, namely O1 and O2, where O1 shares 6 bonds and O2 shares 5 bonds. Different bond lengths between the atoms present in the SZO bulk unit cell are denoted by the terms $\hbox {L}_{1-20}$ in Table 1. To determine the bond lengths (in Table 1) between the atoms in both conventional SZO and SZO with O vacancy, we have used the relaxed and optimized structures from DFT calculations to measure distance between two chosen atoms to obtain corresponding bond lengths, using visualisation program XcrySDen^28^. The range of the bond length between Sr and O atoms is 2.04 to 2.64 Å and between Zn and O ranges from 1.99 to 2.127 Å.

**Table 1:** Structural parameters of both relaxed conventional SZO and SZO with 12.5% O vacancy.

Type of bond	Conventional		12.5% O vacancy		Change (%)
	Bond	Length (Å)	Bond	Length (Å)	
Sr–O	$\hbox {L}_4$	2.614	$\hbox {R}_4$	2.58	$-$ 1.09
	$\hbox {L}_5$	2.60	$\hbox {R}_5$	2.56	$-$ 1.4
	$\hbox {L}_8$	2.60	$\hbox {R}_2$	2.58	$-$ 0.8
	$\hbox {L}_9$	2.59	$\hbox {R}_7$	2.67	2.98
	$\hbox {L}_{{11}}$	2.59	$\hbox {R}_{{11}}$	2.59	–
	$\hbox {L}_{{12}}$	2.60	$\hbox {R}_{{12}}$	2.58	$-$ 0.77
	$\hbox {L}_{{16}}$	2.60	$\hbox {R}_{{16}}$	2.53	$-$ 2.65
	$\hbox {L}_{{17}}$	2.614	$\hbox {R}_{{14}}$	2.54	$-$ 2.83
	$\hbox {L}_{{19}}$	2.64	$\hbox {R}_{{17}}$	2.55	$-$ 3.4
	$\hbox {L}_3$	2.64	–	–	–
Zn–O	$\hbox {L}_1$	2.127	$\hbox {R}_1$	2.30	8.27
	$\hbox {L}_6$	2.127	$\hbox {R}_{{6}}$	2.13	$-$0.66
	$\hbox {L}_7$	1.99	$\hbox {R}_3$	1.99	–
	$\hbox {L}_{{10}}$	2.037	$\hbox {R}_{{10}}$	2.04	0.147
	$\hbox {L}_{{13}}$	1.99	$\hbox {R}_{{9}}$	1.98	$-$ 0.50
	$\hbox {L}_{{14}}$	2.037	$\hbox {R}_{{8}}$	2.00	$-$ 1.81
	$\hbox {L}_{{15}}$	2.127	$\hbox {R}_{{13}}$	2.29	7.66
	$\hbox {L}_{{18}}$	2.127	$\hbox {R}_{{15}}$	2.20	3.5
	$\hbox {L}_{{20}}$	2.037	$\hbox {R}_{{18}}$	2.05	0.63
	$\hbox {L}_{{2}}$	2.037	–	–	–

Different types of bonds and corresponding lengths of conventional and O vacancy cases are represented by $\hbox {L}_{1-20}$ and $\hbox {R}_{1-18}$ in Fig. 1b, c, respectively. Percentage change in the bond lengths due to O vacancy is tabulated in the last column of the table. Negative values denote reduction in the bond length and increase in the length is denoted by positive values.

**Figure 2:**
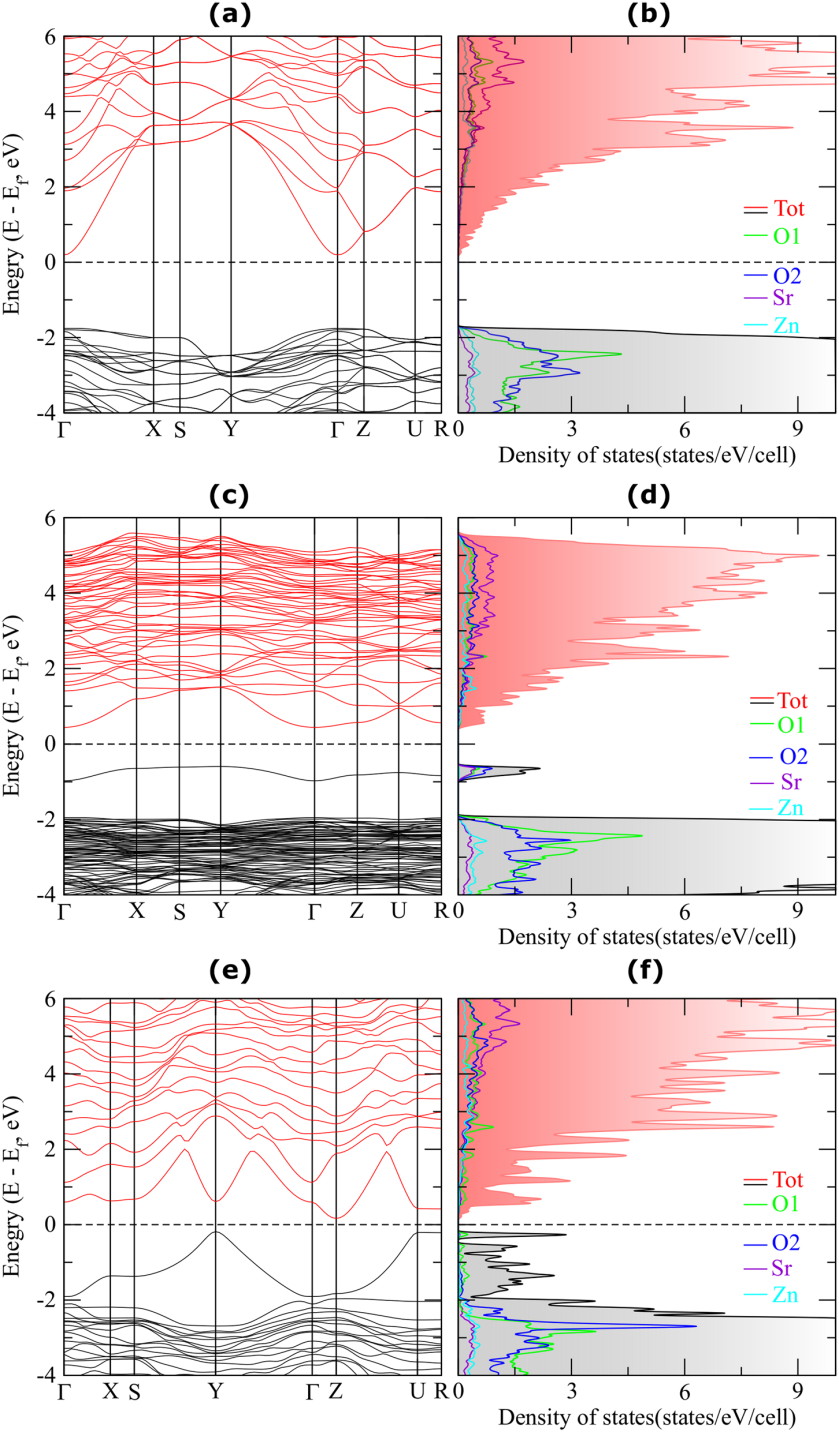
(**a**) Electronic band structure and (**b**) density of states of pristine SZO. (**c**) Electronic band structure and (**d**) density of states of SZO with 3.125% of O vacancy concentration. (**e**) Electronic band structure and (**f**) density of states of SZO with 12.5% of O vacancy concentration. The Fermi level is at zero energy, marked by dashed line. Red shaded region corresponds to the total DOS for conduction bands above the Fermi energy level, whereas, grey shaded region corresponds to the total DOS for valence bands immediately below the Fermi level. Legends in panels **b**,**d** and **f** correspond to respective partial densities of states.

Electronic band structure (Fig. 2(a)) predicted a direct band gap value of 1.95 eV at high symmetry $\Gamma$ point, which is less than experimentally reported band gap^29^. It is evident from the density of states plot (Fig. 2(b)) that contribution to the valence band maximum (VBM) near the Fermi level comes from oxygen orbitals. However, Sr and Zn orbitals are dominated at the conduction band minimum (CBM).

To observe the effect of oxygen vacancy in SZO system, we introduced two types of O vacancy concentrations, (a) by removing single O atom from 16 formula units (from $2 \times 2\times$1 supercell) resulting in 3.125% vacancy concentration, and (b) by removing single O atom from 4 formula units, creating 12.5% vacancy concentration. When an O vacancy is introduced in the system, a prominent reorganization of the bonds occur^30^. For instance, comparing the bonds depicted in Fig. 1(c) with bulk unit cell (Fig. 1(b)), a variation in bond lengths was observed as tabulated in Table 1. The bonds $\hbox {L}_2$ and $\hbox {L}_3$ are present in Fig. 1(b), but missing in Fig. 1(c). The bond length between Sr and O ranges from 2.53 to 2.67 Å and 1.98 to 2.30 Å between Zn and O atoms. Percentage change (increase or decrease) in bond lengths due to oxygen vacancy is shown in the last column of the Table 1. Significant change in the bond lengths can be observed in vicinity of oxygen vacancy. As an example, one of the bond lengths near O vacancy has changed by 8.27% (Table 1).

Due to incorporation of oxygen vacancies in SZO system, the consequent break in intrinsic symmetry is observed^31,32^ which is reflected in shifting of bands in the defect structure, shown in Fig. 2(c) and (e). With introduction of defects, for both 3.125% and 12.5% defect concentration, VBM shifted from $\Gamma$ point in pristine to Y point (see Fig. 2(c), (e) and Brillouin zone in Fig. 1(d)). Likewise, CBM shifted from $\Gamma$ point in pristine to Z point in vacancy structure (Fig. 2(e)). Thus, with oxygen vacancies in SZO, we encountered indirect band gaps (Fig. 2(c) and (e)). By comparing the band structures (Fig. 2(a), (c) and (e)), it can be seen that oxygen vacancies produce new energy states in forbidden gap. With introduction of small concentration of O vacancy (3.125%), the system becomes more electron rich and behaves like *n*-doped system (due to additional unpaired electrons). A distinct impurity band now appeared below the Fermi level, resulting in prominent reduction of band gap to 1.02 eV. Accordingly, a new peak appeared in the corresponding densities of states (Fig. 2(d)) just below the Fermi level. The partial density of states (PDOS, as described by legends with atom types in Fig. 2(b), (d), and (f)) is calculated by projecting the plane wave states on localized orbitals of different shells and angular momentum of corresponding atoms. In our calculation for O vacancy cases, O 2*p* orbitals show maximal contribution to the defect states in the forbidden gap of SZO, pointing to the fact that impurity levels induced by oxygen vacancies originate mainly from O 2*p* orbitals (see PDOS in Fig. 2). With increase in oxygen vacancy concentration (in going from 3.125 to 12.5%), defect-defect interaction becomes more prominent and results in larger dispersion of bands, thereby broadening the density of states corresponding to O vacancy (Fig. 2(e)). Hence, it can be stated from band structure analysis that oxygen vacancies produce new energy states between conduction and valence bands.

In order to understand the relative formation of different O vacancy concentrations, we calculated formation energies ($\hbox {E}_{\mathrm{form}}=\hbox {E}_{\mathrm{(O-vacancy)}} - \hbox {E}_{\mathrm{(perfect)}} +\mu _{\mathrm{O}}$) corresponding to two different O vacancy concentrations in our study. In this definition, $\hbox {E}_{\mathrm{form}}$, $\hbox {E}_{\mathrm{(O-vacancy)}}, \hbox {E}_{\mathrm{(perfect)}}$ correspond to formation energy, supercell energy with single O vacancy, and the pristine supercell energy, respectively, while $\mu _{\mathrm{O}}$ refers to the chemical potential of O which is half of the total energy of an isolated oxygen molecule $\hbox {O}_2$^33^. We found that for 3.125% O vacancy concentration, $\hbox {E}_{\mathrm{form}}$ = 4.1 eV, while for 12.5% O vacancy concentration, $\hbox {E}_{\mathrm{form}}$ = 4.7 eV. These trends in formation energies and the relative stabilities of different O vacancy concentrations were comparable to what reported in other oxide system^33^. Note that in many semiconductors, charged O vacancy is known to be thermodynamically stable and thus energetically feasible, more so in *n*-type conductive systems. For example, Liu et al.^34^ reported that + 2 charged states are possible in O vacancies in ZnO systems, and depends delicately on chemical potential, Fermi level, overall oxygen self-diffusion within ZnO heterostructures. Another recent study of O vacancies in molybdenum oxide semiconductor^35^ suggested that oxygen vacancy formation in this material leaves two electrons on the surface, and generates two different configurations: either two $\hbox {Mo}^{5+}$ centers or a single double-reduced $\hbox {Mo}^{4+}$, thereby diffusing the charged state from O to Mo atoms. Pham et al.^36^ reported formation of O vacancies with neutral, + 1 and + 2 states in amorphous and rutile phase of titanium dioxide, depending strongly on the Fermi energy of the system. Their simulations suggested that at Fermi levels near the valence band maxima, the positively charged + 1 and + 2 O vacancy states could form spontaneously in amorphous $\hbox {TiO}_2$, while with increase in the Fermi energy, formation energy of the positively charged defect increases. Thus, the study of O vacancy diffusion and charged states of O vacancies in SZO and systems alike demands dedicated research efforts through experiments and simulations.

**Figure 3:**
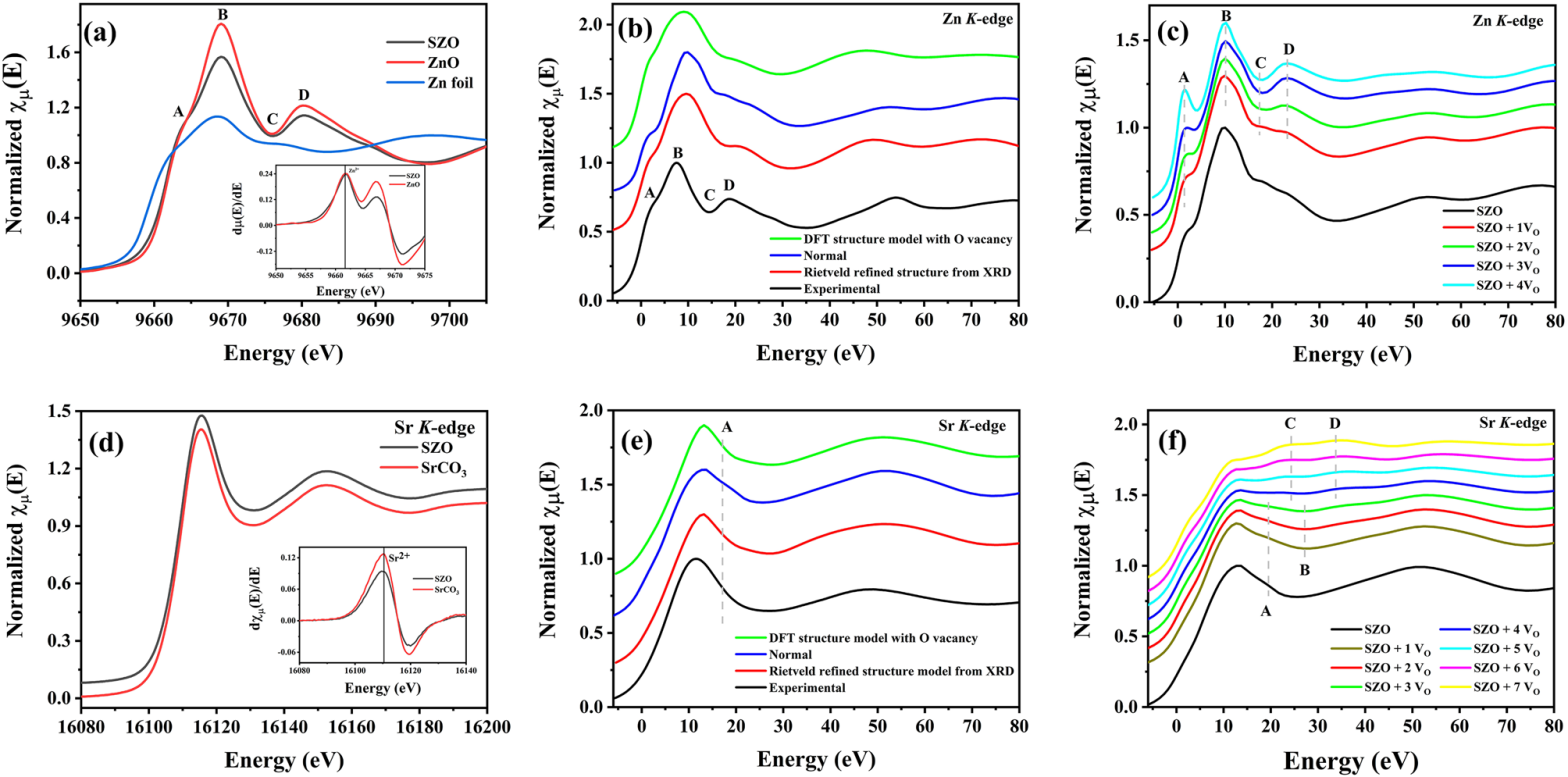
(**a**) Zn *K*-edge experimental XANES spectrum for SZO along with reference spectra of ZnO and Zn foil. Inset showing the first derivative of absorption coefficient for SZO and ZnO. (**b**) Experimental and simulated XANES at Zn *K*-edge for normal, Rietveld refined and 12.5% oxygen vacancy incorporated DFT optimised structure model. (**c**) Simulated XANES for SZO system in bulk as well as vacancy mediated configuration with successive removal of oxygen atoms around Zn core in first shell, where $\hbox {V}_{\mathrm{O}}$ indicates oxygen vacancy. (**d**) Sr *K*-edge experimental XANES spectrum for SZO along with reference spectrum of $\hbox {SrCO}_3$. Inset showing the first derivative of absorption coefficient for SZO and $\hbox {SrCO}_3$. (**e**) Experimental and simulated XANES at Sr *K*-edge for normal, Rietveld refined and 12.5% oxygen vacancy incorporated DFT optimised structure model. (**f**) Simulated XANES for SZO system in bulk as well as vacancy mediated configuration with successive removal of oxygen atoms around Sr core in first shell, where $\hbox {V}_{\mathrm{O}}$ indicates oxygen vacancy. $\hbox {E}_0$ of all the simulated XANES is set to zero and curves are stacked vertically for comparison.

After seeing the effect of oxygen vacancies on band structure, its impact on local electronic structure was probed through XAS study at Zn and Sr *K*-edges, as shown in Fig. 3. Talking at first about Zn *K*-edge, the normalized XANES mapping the 1*s* $\rightarrow$ 4*p* transitions^37,38^ is represented in Fig. 3(a), along with reference absorption *K*-edges of Zn foil and ZnO. The white line is not fully coinciding with the absorption edge for + 2 oxidation state (ZnO), but at slight low energy than that of ZnO. The first derivative of absorption coefficient gives edge energy ($\hbox {E}_0$) of 9661.7 eV and 9661.9 eV for SZO and ZnO, respectively, indicating 0.2 eV red shift of $\hbox {E}_0$ in SZO. The features obtained at 9663.6 eV, 9669.1 eV, 9676.1 eV and 9680.3 eV are marked as A, B, C and D, respectively. It is to be noted that comparable features were also obtained previously in SZO prepared by combustion synthesis using monoethanolamine (MEA) as fuel, where extended X-ray absorption fine structure (EXAFS) analysis revealed presence of oxygen vacancies in that system. Howbeit, denomination of various features was not clear^1^.

Comparing the Zn *K*-edge XANES in SZO with that in ZnO, one could see that signal is very much alike in profile, apart from variation in intensity of features. The defect imprints on XANES in ZnO are reported with various flavours via experimental and theoretical approaches^39^. Hsu et al.^40^ correlated the pre-edge feature in first derivative of XANES as signature of oxygen vacancies in the system in Ar/$\hbox {H}_2$ annealed Co doped ZnO. They recreated the similar XANES by adding oxygen vacancy in the system through real space multiple scattering simulations. Whereas, Zhang et al.^41^ created oxygen vacancies in the system through Ar annealing and reported the decrease in intensity of absorption edge as mark of oxygen vacancy, unlike any pre-edge feature claimed by Hsu et al.^40^. Similarly, the results shown by Guglieri et al.^42^ and Haug et al.^43^ after considering oxygen vacancies in ZnO based system was found to be affecting whole of the XANES spectrum, with final result contrary to each other as well with that shown by Hsu et al.^40^. As the XANES in pure SZO system is not reported previously to explain the variation with intrinsic defects, this posed the need to perform vacancy incorporated XANES simulation for SZO system and to get any evidence about agreement (if any) with what reported in ZnO system.

Thus, the theoretical XANES analysis was commenced with simulation of normal structure model (without vacancy) and oxygen vacancy incorporated DFT optimized structure model (12.5% vacancy). Rietveld refined structure from X-ray diffraction analysis^16^ was also used to add flavour of experimentally procured structure derived simulated signal. The simulated XANES for these structure models along with experimental spectrum is represented in Fig. 3(b), with features labelled as per Fig. 3(a). It can be perceived that the simulated XANES for ideal crystal structure of SZO system is not fully reproducing the experimental spectrum. Apart from mismatch in position of feature A, a big difference is seen in feature C and D which were mistaken to be mimicking Zn-O environment as in ZnO system. Similarly, XANES for oxygen vacancy incorporated DFT optimised structure is showing some variation than XANES for ideal crystal structure of SZO. The features are slightly broad but the minute changes in marked features can be easily visualized. The represented scan is shown after averaging the simulated signal for all four zinc atoms of single unit cell. During derivation of XANES from vacancy model, the averaged signal is having contribution from core atoms, with and without oxygen vacancy in their vicinity, as shown in Fig. S1 of Supplementary file and the observed broad feature is justified due to involvement of absorbers with varied environments, resulting in varied signals. The vacancy structure saw upward shift of feature A as compared to its position in normal structure. But the overall intensity of features is less pronounced as in experimental scan. On the other hand, XANES signal from Rietveld refined structure is having appreciable change in feature C and D. These evolved features in experimental structure are expected due to presence of enhanced concentration of various vacancies/defects in the system. The comparison of experimental signal with these scans anticipated the altering of C and D features due to presence of defects/vacancies in the system.

In next step, for elaborative decryption of the features obtained in experimental spectrum, theoretical models having diverse oxygen vacancies around Zn absorber are tested for their effect on Zn *K*-edge, as is shown in Fig. 3(c). The oxygen atoms around Zn were removed one by one from input file without using any adjustable parameter to compensate for vacancy. Our aim behind this simulation was to observe the envisaged modifications in XANES on introducing oxygen vacancies in the system. Figure 3(c) shows systematic change in marked features on removing oxygen atoms around Zn in SZO. The dip in feature C and rise in feature D is in good agreement with experimentally obtained XANES, indicating that mismatch in experimental and theoretical signal is due to effect of oxygen vacancies around Zn atoms. The shoulder feature A is rising in intensity with increase in oxygen vacancy and is not responding well to the experimental feature. As observed in various reports in ZnO system, the behaviour of feature A in experimental samples is significantly impacted by state of sample (bulk, nano or thin films)^39,43,44^. The non-compliance of this feature in theoretical and experimental scan can be attributed to structural effects. Thus, the rise in XANES at 9680.3 eV at Zn *K*-edge (Fig. 3(a)) in SZO is the mark of oxygen vacancies on its local electronic structure.

Similarly, XANES at Sr *K*-edge, mapping 1*s* $\rightarrow$ 5*p* transitions, is shown in Fig. 3(d) along with reference scan of $\hbox {SrCO}_3$. The XANES of SZO and $\hbox {SrCO}_3$ are quite identical, due to which two signals are shown at y-offset. The first derivative of absorption coefficient (inset Fig. 3(d)) also indicates that $\hbox {E}_0$ value, 16110.6 eV, is eclipsing the + 2 oxidation state of Sr. On sieving the literature, it was observed that most of Sr *K*-edge signals in diverse compounds exhibit similar traits, as shown in Fig. 3(d)^45,46^. Comparable XANES was also observed in SZO prepared by MEA fuel in our previous study, which was having oxygen vacancies in the system^1^. Also, the reference scan ($\hbox {SrCO}_3$), shown in Fig. 3(d), is reported to be having 9 atoms in first coordination shell^47^, while Sr in SZO have 7 atoms, which is creating hurdle in hypothesizing Sr environment in SZO.

Similar theoretical approach was employed for Sr *K*-edge, as was used in Zn *K*-edge, to compare the experimental XANES with theoretical XANES derived from various structure models, shown in Fig. 3(e). It is apparent that experimental Sr *K*-edge is showing good agreement with XANES obtained from Rietveld refined structure and oxygen vacancy incorporated DFT optimized structure. Whereas, structure model with no vacancy (normal structure) is having shoulder peak post absorption edge, marked as A, which is absent in other scans. While commencing with XANES simulation, varying full multiple scattering (FMS) clusters were worked out to get final cluster yielding converging results, shown in Fig. S2 of supplementary file. It was observed that small FMS clusters (upto 4.80 Å) were not having the suspected feature, whereas, FMS cluster beyond 4.80 Å started sprouting this feature. Also, appreciably good signal of $k^2$ weighted EXAFS function upto 10 ${\AA }^{-1}$ pointed towards fairly well crystallinity of the sample (See Supplementary Fig. S3). So, the comparison ushered to the fact that presence of feature A is expected in theoretical and experimental Sr *K*-edge XANES. According to literature, feature A at Sr *K*-edge is usually ascribed to single and multiple scattering contributions from distant atoms^48^ or indicative of high coordination number of strontium^49^. Hence, absence of feature A can be assimilated as mark of vacancies around Sr absorber in SZO.

Imitating the steps taken in Fig. 3(c), XANES is simulated by successively removing oxygen atoms one by one around Sr absorber to know the modifications in XANES (shown in Fig. 3(f)). The feature of interest A is found to be diminishing with increasing oxygen vacancies. Whereas, choking the first coordinate shell around Sr with oxygen vacancies lead to bifurcation of feature B into C and D. Apart from marked features, a hump on absorption edge also starts emerging on higher vacancy side. The peculiar evolution of XANES with vacancies is not exactly observed in experimental scan, but absence of feature A at low vacancy concentration supported the fact forged by analysing Fig. 3(e). Hence, we could say that absence of feature A is mark of oxygen vacancies around Sr absorbed in SZO.

The first coordination shell of Fourier transformed EXAFS for Zn and Sr in SZO is dominated by single scattering paths from oxygen atoms. The second shell contains contributions from scattering paths from neighbouring Zn and Sr atoms for both absorbers. Thus, to get information about coordination environment around Zn and Sr, the Fourier transformed (FT) EXAFS function for Zn and Sr *K*-edges were fitted against theoretical SZO model using ARTEMIS software (version 0.9.26, url: https://bruceravel.github.io/demeter/)^50^, shown in Figs. S4 and S5 of Supplementary Material. The details about paths and parameters used in fitting is given in section S1 of Supplementary Material. The *k*-range of 2.5-12 ${\AA }^{-1}$ and 3-8 ${\AA }^{-1}$ was used for FT of Zn and Sr *K*-edges EXAFS data, respectively. The EXAFS fittings were performed in phase uncorrected R-space range of 1-3.44 Å for both edges. The parameters extracted from the fit are listed in Supplementary Table S1. It can be seen that coordination number for Zn-O1, Zn-O2 and Zn-O1 came out to be 0.9, 1.8 and 0.9 with respective bond lengths of 1.91, 1.95 and 1.99 Å. Similarly, coordination number for Sr-O2, Sr-O1 and Sr-O2 came out to be 3.7, 1.6 and 0.8 with respective bond lengths of 2.52, 2.56 and 2.70 Å. The decreased oxygen coordination around Zn and Sr also indicates the presence of oxygen vacancies in the system. The second shell EXAFS analysis, having metal-metal bonds, also indicated about decreased coordination number and variation in bond distances of respective paths. Thus, presence of oxygen vacancies in the system is impacting bonding structure of the material, as is also observed from Table 1 and variation in DOS with addition of oxygen vacancy in the system.

**Figure 4:**
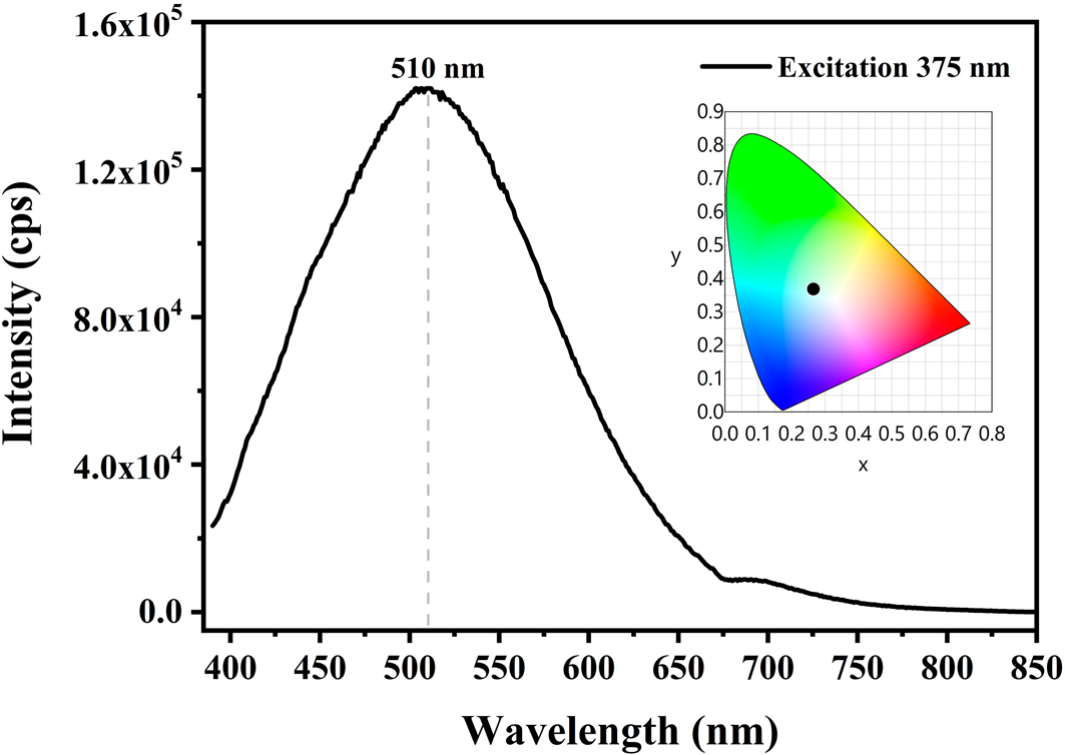
Photoluminescence spectrum of SZO nanophosphors upon excitation at 375 nm, inset having CIE coordinate diagram for corresponding emission.

After evidencing emergence of new energy states in forbidden gap, analysing modification of XANES features at Zn and Sr *K*-edges and decreased oxygen coordination number around Sr and Zn absorbers, the oxygen vacancies are expected to contribute in PL emission from SZO nanophosphors. A broad PL emission spanning in range 400-600 nm and centering $\sim 510\hbox { nm}$ is observed at excitation upon 375 nm, shown in Fig. 4. The overall visible emission falls in white region (inset of Fig. 4). No band to band emission is observed in PL spectrum, which indicates the role of defect states present in forbidden gap responsible for the emission. Recently, our group reported that SZO nanophosphors synthesized by combustion synthesis using MEA fuel exhibit broad emission centering $\sim 510\hbox { nm}$ upon below band gap excitation bestowed by oxygen vacancies^1^. The slight change in emission is expected due to variation in synthesis conditions. Apart from this, similar emission trends in ZnO system at below band gap excitation are reported and discussed heavily supported by various mechanisms^51–55^. This green centred emission is ascribed to transition between shallow defect states and oxygen vacancy induced deep defect states^52,55^. The presented band structure and XAS studies recognize deep defect states as consequence of oxygen vacancies in the system. However, the genesis of shallow defect states is currently unknown. Thus, we may here affirm that radiative transitions observed in SZO system are due to electronic transition between shallow defect levels and deep defect levels (generated by oxygen vacancies). On the other hand, nanosized nanoparticles spark yet another possibility of presence of surface defect states due to considerable surface to volume ratio and hence invites further exploration in this system. The radiative emission imparting luminescence in white region proposes SZO as a suitable candidate for white emitting devices and solid state lighting.

## Conclusions

The effect of oxygen vacancies on band structure and local electronic structure of $\hbox {SrZnO}_2$ (SZO) nanophosphors is studied experimentally and theoretically. The combustion synthesized nanophosphors exhibited broad photoluminescence centred at $\sim 510$ nm upon excitation at 375 nm, with overall emission in white region. Having no band to band transition peak but broad spectrum indicated about presence of defect states in forbidden gap, which is also confirmed by the band structure and densities of states analyses, as calculated using density functional theory. The band structure for bulk pristine SZO obtained within generalized gradient approximation estimated the direct band gap of 1.95 eV, while for SZO with oxygen vacancy concentrations 3.125% and 12.5%, the band gaps conceived indirect nature and are substantially reduced. The introduction of oxygen vacancies created new energy states in forbidden gap accompanied by intrinsic symmetry break and hence shifted the valence band maxima and conduction band minima from $\Gamma$ point to Y and Z point, respectively. These defect states are predicted as origin of broad band photoluminescence in SZO nanophosphors. XANES signal at Zn and Sr *K*-edges showed slight mismatch with theoretically simulated XANES. Zn *K*-edge showed varied features at 9676.1 eV and 9680.3 eV and Sr *K*-edge was lacking shoulder peak post absorption edge when matched to theoretical XANES of respective edges. Near edge simulations after successive removal of oxygen atoms around Zn and Sr cores successfully imitated the experimental results, which confirmed the presence of oxygen vacancies in the system. Obtained XANES results would serve as template to be used for sample status judgement in oxygen vacancy state for similar systems. Thus, defect assisted white emission in SZO nanophosphors envisages it as potential candidate for solid state lighting devices.

## Methods

SZO nanophosphors were synthesized by combustion technique using glycine as fuel. The details of synthesis along with X-ray diffraction study are reported somewhere else^16^. PL study of SZO nanophosphors was done on Edinburgh Fluorescence spectrometer (FLS980), equipped with Xenon lamp.

XAS data at Zn and Sr *K*-edges was collected at room temperature in Quick EXAFS mode at Scanning EXAFS beamline (BL-09) of INDUS-2, RRCAT, Indore. This beam line's energy range is 4-25 keV and a typical resolution ($\Delta \hbox {E/E}$) of $10^{-4}$ at 10 keV photon energy has been obtained. The beam line optics mainly consist of an Rh/Pt coated meridional cylindrical mirror, a Si (111) (2d = 6.2709 Å) based double crystal monochromator (DCM) and Rh/Pt coated bendable post mirror. The 0.5 mm $\times$ 0.5 mm (h $\times$ v) size of the beam was obtained at the sample. The ionization chambers (300 mm length each) of suitable gas pressure were used for data collection. The gas mixture have been selected to achieve 10–20% absorption in 1st ionization chamber and 70–90% absorption in 2nd ionization chamber to obtain better signal to noise ratio. For the present samples, XANES and EXAFS measurements were done in transmission mode for the Zn *K*-edge and Sr *K*-edge measurements. The energy range of Zn and Sr edges was calibrated using standard Zn foil and $\hbox {SrCO}_3$ powder, respectively.

To compliment the experimental XANES, theoretical simulations of XANES were performed using FEFF9 software (version 9.6 revision 4, url: http://monalisa.phys.washington.edu/)^56^ over a full multiple scattering (FMS) cluster of $\sim$ 300 atoms. Self consistent field (SCF) was chosen over cluster enclosing 39 atoms (5 Å). All the absorption edges were calculated using Hedin–Lundqvist self energy with screened core hole as per final state rule, with simultaneously running local DOS card to get Fermi level values for each XANES spectra. The details of input parameters for XANES simulations at Zn and Sr *K*-edges is given in section S2 of Supplementary file.

The analysis of atomic and electronic structures of SZO system was carried out using DFT, where the properties of a many-electron system can be determined using spatially dependent electron density obtained from the self-consistent iterative solutions of Kohn-Sham equations^57^. Here, we employed norm-conserving pseudo-potential for the representation of the valence states within generalized gradient approximation (GGA) scheme as proposed by Perdew, Burke, and Ernzerhof (PBE) for the exchange and correlation energy^58^, as implemented in quantum espresso (QE) package^59^. These choices of pseudopotentials have been tested to be appropriate for low dimensional materials in general^60–63^. While performing Heyd–Scuseria–Ernzerhof (HSE) hybrid functional calculations are known to provide better band gap, HSE band gaps actually scales linearly with PBE band gaps^64^. Hence, PBE results may act as efficient descriptors for more expensive HSE calculations, as well as the trends suggested by the experimental results. We tested the convergence of total energy of electrons with respect to *k*-points, energy cut-off and other important parameters and finally chose a plane-wave basis set with an energy cut-off of 80 Ry. The crystal structure was fully relaxed until the final force exerted on each atom reaches below 0.001 Ry/Bohr and an electronic energy convergence threshold of $10^{-4}$ Ry was achieved. For the Brillouin zone integration, we used the *k*-point sets generated by the $12\times 12 \times 8$ Monkhorst–Pack^65^ *k*-point mesh. Electronic bands were plotted along the high symmetry directions $\Gamma \rightarrow X \rightarrow S \rightarrow Y \rightarrow \Gamma \rightarrow Z \rightarrow U \rightarrow R$. The crystal structure of SZO was simulated with an in-plane rectangular unit cell.

## Supplementary information

Supplementary information.
