# Supplementary material for: Oxygen vacancies induced photoluminescence in SrZnO2 nanophosphors probed by theoretical and experimental analysis
Source: Sci Rep. 2020 Oct 15;10:17364. doi: 10.1038/s41598-020-74436-8 (PMC7567121; doi:10.1038/s41598-020-74436-8)
Supplement: Supplementary file 1 — Supplementary information. [file 41598_2020_74436_MOESM1_ESM.pdf]

Oxygen vacancies induced photoluminescence in $SrZnO_2$ nanophosphors probed by theoretical and experimental analysis

Manju[1,2], Megha Jain[1,2], Saibabu Madas[3,4], Pargam Vashishtha[5,6], Parasmani Rajput[7], Govind Gupta[5], Mousumi Upadhyay Kahaly[3,4], Kemal Özdoğan[8], Ankush Vij[9,*] and Anup Thakur[1,#]

[1]*Advanced Materials Research Lab, Department of Basic and Applied Sciences, Punjabi University, Patiala-147 002, Punjab, India*
[2]*Department of Physics, Punjabi University, Patiala-147 002, Punjab, India*
[3]*ELI-ALPS, ELI-HU Non-Profit Ltd., Dugonics tér 13, Szeged 6720, Hungary*
[4]*Institute of Physics, University of Szeged, Dóm tér 9, H-6720, Szeged, Hungary*
[5]*Sensor Devices & Metrology Group, CSIR- National Physical Laboratory (CSIR-NPL), Dr. K. S. Krishnan Road, New Delhi-110 012, India*
[6]*Academy of Scientific & Innovative research, (AcSIR), CSIR-HRDC campus, Ghaziabad-201 002, Uttar Pradesh, India*
[7]*Atomic & Molecular Physics Division, Bhabha Atomic Research Center, Trombay, Mumbai-400 085, India*
[8]*Department of Physics, Yildiz Technical University, 34210 Istanbul, Turkey*
[9]*Nanophosphors Lab, Department of Physics, Amity University Haryana, Gurgaon-122 413, Haryana, India*
[*]*Email:* vij_anx@yahoo.com
[#]*Email:* dranupthakur@gmail.com

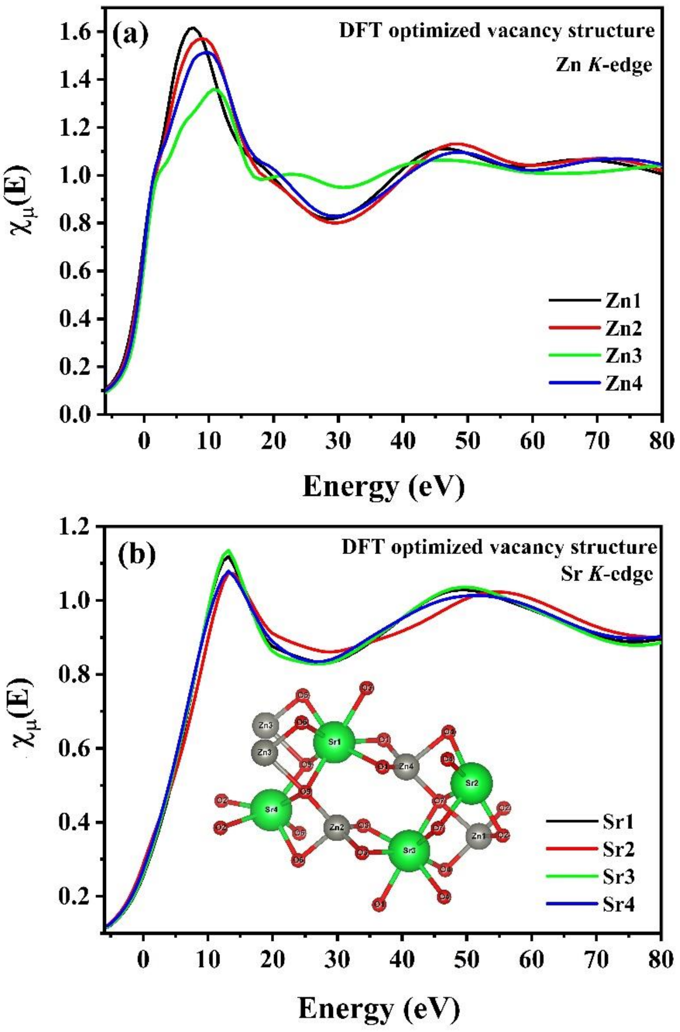

Figure S1: Simulated XANES for DFT optimized structure having oxygen vacancy at (a) Zn *K*-edge and (b) Sr *K*-edge. The inset of (b) is showing the relaxed unit cell after adding oxygen vacancy into it.

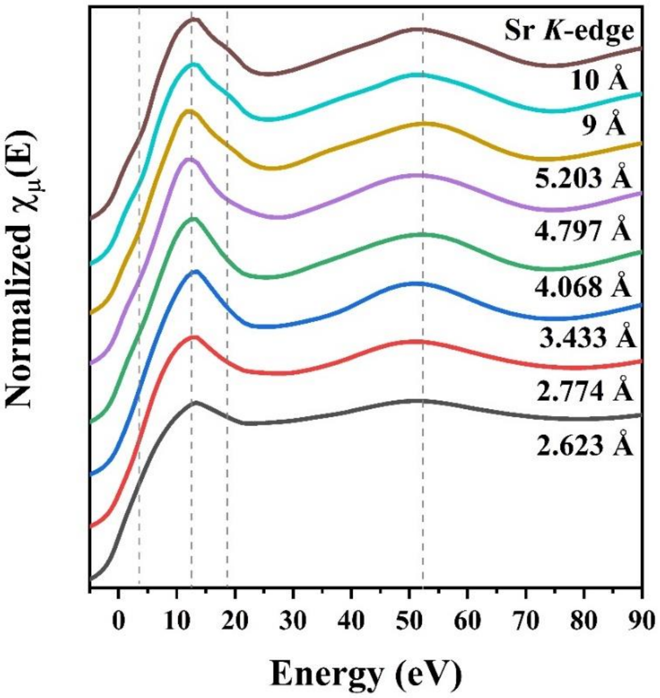

Figure S2: Effect of increasing full multiple scattering cluster for XANES simulation around Sr absorber.

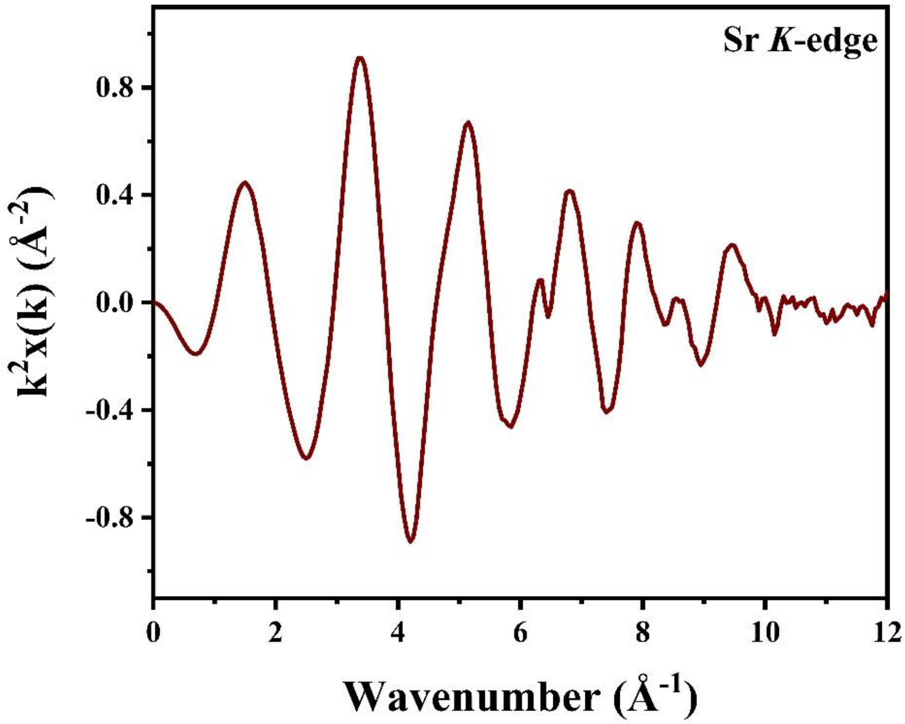

Figure S3: k^2 weighted EXAFS function for Sr *K*-edge of SZO nanophosphors.

Section S1:

Sample preparation and data collection:

For XAS measurement, the sample (30 mg for Zn *K*-edge and 50 mg for Sr *K*-edge) was thoroughly mixed with cellulose (total weight 0.1 g), to obtain an edge jump of 1. The pallets of 15 mm diameter for formed for both the edges. Thus, for Zn and Sr *K*-edges, 12 and 20 scans were merged to get final spectra. The time resolution of measured data was ~150 ms.

EXAFS data analysis:

The standard procedures [1,2] were followed in analysis of EXAFS data using IFEFFIT software package (version 0.9.26, url: https://bruceravel.github.io/demeter/) [3]. The energy dependent absorption coefficient $\mu(E)$ was converted into energy dependent absorption function $\chi(E)$, which was subsequently converted into wave number dependent absorption coefficient $\chi(k)$. After that, k^2 weighted $\chi(k)$ was Fourier transformed into $\chi(R)$, to generate signal *versus* R i.e. in terms of real distance from the absorbing centres. These steps were performed in ATHENA subroutine. The theoretical EXAFS spectrum was generated from assumed crystallographic structure, in ARTEMIS subroutine employing ATOMS and FEFF6.0 code [4,5]. The experimental data was fitted against this theoretical model and goodness of fit was observed by R_{factor}, which is defined in equation (1),

$$R_{factor} = \sum \frac{[\mathrm{Im}(\chi_{dat}(r_i) - \chi_{th}(r_i)]^2 + [\mathrm{Re}(\chi_{dat}(r_i) - \chi_{th}(r_i)]^2}{[\mathrm{Im}(\chi_{dat}(r_i)]^2 + [\mathrm{Re}(\chi_{dat}(r_i)]^2} \quad \text{---------------- (1)}$$

where, χ_{dat} and χ_{th} refer to the experimental and theoretical $\chi(R)$ values, respectively and *Im* and *Re* refer to the imaginary and real parts of the respective quantities, respectively.

Fig. S4 and S5 show the Fourier transformed (FT) EXAFS functions for Zn and Sr *K*-edges. The *k*-range of 2.5-12 Å^{-1} and 3-8 Å^{-1} were used for Zn and Sr *K*-edge EXAFS, respectively. The fittings were performed in phase un-corrected *R* space range of 1-3.44 Å for both the edges. For Zn *K*-edge EXAFS fitting, theoretical model of SZO for this absorber was run and relevant paths were chosen for fitting. Three single scattering paths from oxygen atoms i.e. Zn-O1, Zn-O2 and Zn-O1 with theoretical interatomic distances of 1.95 Å, 1.99 Å and 2.03 Å having degeneracy of paths as 1, 2 and 1, respectively, were chosen for first shell. The second shell is having dominant contribution from metal-metal bonds, thus single scattering paths between Zn-Sr1, Zn-Zn1 and Zn-Sr2 were chosen with theoretical interatomic distances of 3.18 Å, 3.35 Å, 3.44 Å having degeneracy of paths as 5, 2 and 2, respectively. Similarly, for Sr *K*-edge EXAFS fitting, three single scattering paths from oxygen atom were chosen as contributors to first shell, which are Sr-O2, Sr-O1 and Sr-O2 with theoretical interatomic distances of 2.58 Å, 2.62 Å and 2.77 Å, having degeneracy of paths as 4, 2 and 1, respectively. Similarly, for second shell, the metal-metal single scattering paths viz. Sr-Zn1, Sr-Sr1 and Sr-Zn2 with theoretical interatomic distances of 3.18 Å, 3.35 Å and 3.44 Å were chosen, having degeneracy of 5, 2 and 2, respectively. Regarding radial distance correction term, single parameter for first shell and different parameters for second shell paths were chosen. The Debye Waller factors and correction factor for coordination number were different for all the paths. The results after fitting are tabulated in tab. S1.

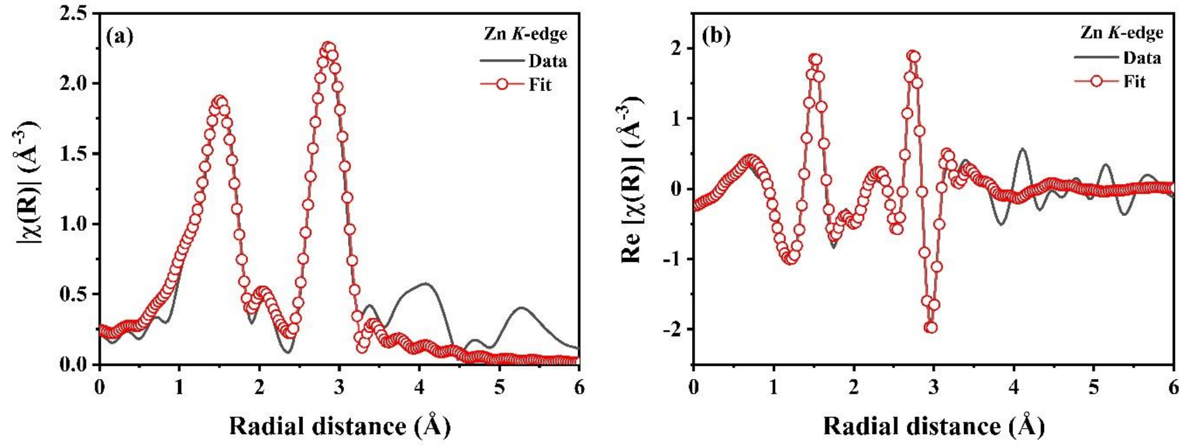

Figure S4: (a) and (b) represents magnitude and real part of Fourier transformed EXAFS function for Zn *K*-edge. (The Fourier transformed curves showed here are phase uncorrected).

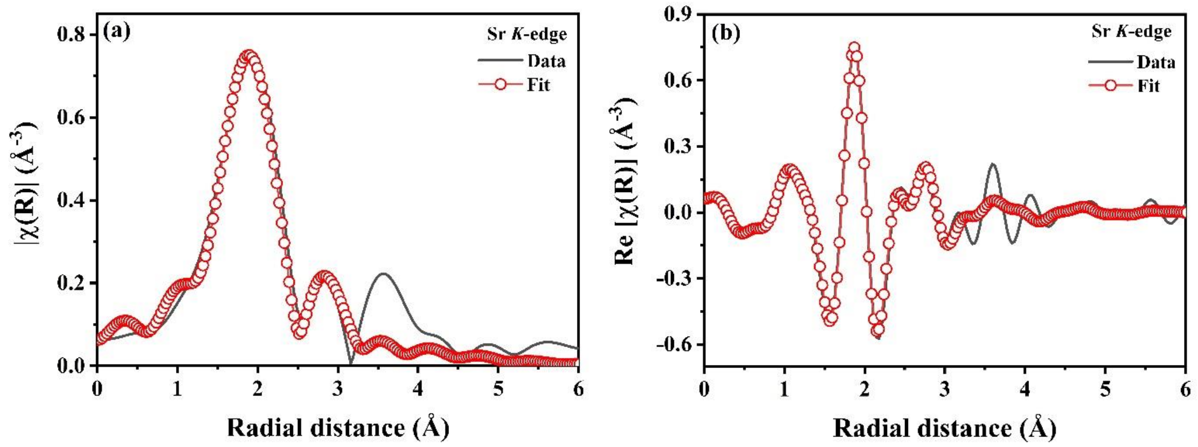

Figure S5: (a) and (b) represents magnitude and real part of Fourier transformed EXAFS function for Sr *K*-edge. (The Fourier transformed curves showed here are phase uncorrected).

Table S1: Coordination number (*N*), interatomic distance (*R*) and Debye Waller factor (σ^2) obtained by EXAFS analysis of first coordination shell for Zn and Sr *K*-edges. The amplitude reduction factor (S_0^2)* was taken from fitting of ZnO and $SrCO_3$ for Zn and Sr *K*-edges, respectively. The numbers in parentheses indicate the uncertainty in the last digit.

	**Paths**	**N**	**R (Å)**	**σ^2 (Å^2)**
**Zn K-edge**	Zn-O1	0.9 (2)	1.91 (2)	0.0041 (3)
	Zn-O2	1.8 (3)	1.95 (2)	0.0034 (2)
	Zn-O1	0.9 (4)	1.99 (2)	0.0046 (3)
	Zn-Sr1	4.9 (2)	3.21 (3)	0.0125 (4)
	Zn-Zn	1.8 (2)	3.24 (4)	0.0049 (4)
	Zn-Sr2	1.9 (3)	3.22 (4)	0.0067 (4)
**Sr K-edge**	Sr-O2	3.7 (2)	2.52 (2)	0.0055 (3)

	Sr-O1	1.6 (2)	2.56 (3)	0.0062 (3)
	Sr-O2	0.8 (2)	2.70 (2)	0.0062 (3)
	Sr-Zn1	3.7 (2)	3.21 (3)	0.0136 (3)
	Sr-Sr	1.5 (2)	3.28 (4)	0.0096 (3)
	Sr-Zn2	1.5 (3)	3.44 (3)	0.0119 (3)

* The amplitude reduction factors obtained from fitting of ZnO and $SrCO_3$ are 1.05 ± 0.08 and 0.99 ± 0.04, respectively, whereas R-factor is 0.01 for both edges.

Section S2: XANES simulation details:

The input file for FEFF9 software to run XANES simulations was generated from ATOMS software [4]. Optimized cluster for self-consistent field (SCF) and full multiple scattering (FMS) were chosen till converging results. All the scans were simulated after accounting for core-hole broadening and instrumental broadening (1.7 eV for Zn *K*-edge and 3 eV for Sr *K*-edge). Thus, the input file for both Zn and Sr *K*-edge XANES is shown as follows:

FEFF Input file:

Edge K

SO2 1.0

CONTROL 1 1 1 1 1 1

SCF 5.0 0 100 0.2 1

LDOS -30 50 0.1

COREHOLE FSR

XANES 10.0 0.06 0.1

FMS 8.0 0

POTENTIALS

Ipot Z tag

0 20 Zn 2 2 0.001	0 38 Sr 3 3 0.001
1 8 O 1 1 1.0	1 8 O 1 1 1.0
2 30 Zn 2 2 1.0	2 30 Zn 2 2 1.0
4 38 Sr 3 3 1.0	4 38 Sr 3 3 1.0

Further information about FEFF9 simulations and parameter terminology can be gained from http://monalisa.phys.washington.edu/index-feffproject.html

References:

1. Konigsberger, D.C, Prince, R. X-ray Absorption: Principles, Application, Techniques of EXAFS, SEXAFS and XANES; Wiley, New York (1988).
2. Ulery, A. and Drees, R. Methods of Soil Analysis: Part 5-Mineralogical Methods; Wiley Online Library (2008).
3. Ravel, B., Newville, M. ATHENA, ARTEMIS, HEPHAESTUS; Data Analysis for X-ray Absorption Spectroscopy using IFEFFIT, J. Synchrotron Radiat. 12, 537-541 (2005).
4. Zabinsky, S.I., Rehr. J.J., Ankudinov, A., Albers, R.C., Eller, M.J. Multiple-scattering calculation of X-ray absorption spectra, Phys. Rev. B 52, 2995-3009 (1995).
5. Ravel, B. ATOMS: crystallography for the X-ray absorption spectroscopist, *J. Synchrotron Radiat.* **8**, 314-316 (2001).
